# Vertical Distribution of Bacterial Community Diversity and Water Quality during the Reservoir Thermal Stratification

**DOI:** 10.3390/ijerph120606933

**Published:** 2015-06-17

**Authors:** Hai-Han Zhang, Sheng-Nan Chen, Ting-Lin Huang, Wei-Xing Ma, Jin-Lan Xu, Xin Sun

**Affiliations:** School of Environmental and Municipal Engineering, Xi’an University of Architecture and Technology, No.13, YanTa Road, Xi’an 710055, China; E-Mails: chenshengnan@xauat.edu.cn (S.-N.C.); huangtinglin@xauat.edu.cn (T.-L.H.); hs_weixing@163.com (W.-X.M.); xujinlan@xauat.edu.cn (J.-L.X.); sunxin@xauat.edu.cn (X.S.)

**Keywords:** reservoir, thermal stratification, water bacterial community, high-throughput sequencing

## Abstract

Reservoir thermal stratification drives the water temperature and dissolved oxygen gradient, however, the characteristic of vertical water microbial community during thermal stratification is so far poorly understood. In this work, water bacterial community diversity was determined using the Illumina Miseq sequencing technique. The results showed that epilimnion, metalimnion and hypolimnion were formed steadily in the JINPEN drinking water reservoir. Water temperature decreased steadily from the surface (23.11 °C) to the bottom (9.17 °C). Total nitrogen ranged from 1.07 to 2.06 mg/L and nitrate nitrogen ranged from 0.8 to 1.84 mg/L. The dissolved oxygen concentration decreased sharply below 50 m, and reached zero at 65 m. The Miseq sequencing revealed a total of 4127 operational taxonomic units (OTUs) with 97% similarity, which were affiliated with 15 phyla including Acidobacteria, Actinobacteria, Armatimonadetes, Bacteroidetes, Caldiserica, Chlamydiae, Chlorobi, Chloroflexi, Cyanobacteria, Firmicutes, Gemmatimonadetes, Nitrospirae, Planctomycetes, Proteobacteria, and Verrucomicrobia. The highest Shannon diversity was 4.41 in 45 m, and the highest *Chao* 1 diversity was 506 in 5 m. *Rhodobacter* dominated in 55 m (23.24%) and 65 m (12.58%). *Prosthecobacter* dominated from 0.5 to 50 m. The heat map profile and redundancy analysis (RDA) indicated significant difference in vertical water bacterial community composition in the reservoir. Meanwhile, water quality properties including dissolved oxygen, conductivity, nitrate nitrogen and total nitrogen have a dramatic influence on vertical distribution of bacterial communities.

## 1. Introduction

Freshwater microbial community composition plays pivotal roles in sustaining the function and health of drinking water reservoir ecosystems because functional microbes drive several material metabolism and energy conversion pathways that regulate water quality [1,2]. Despite the importance of microbial composition structure in these public health-related aquatic systems, much literature is focused on exploring the sediment, bacterial, or fungal communities harbored in the reservoirs [1,3,4,5] and shallow lakes [6,7]. Compared with the eutrophic lakes, drinking water reservoirs are typically characterized by oligotrophic water with lower carbon, nitrogen and phosphorus loading [8,9]. Unfortunately, information regarding the water bacterial community diversity of drinking water reservoirs is severely limited.

Thermal stratification is a vital physical factor controlling nutrient metabolism and transformation in the drinking water reservoir ecosystem, especially in the deep reservoirs [8,10]. During the stratified periods, epilimnion, metalimnion, and hypolimnion layers are formed steadily in deep reservoirs [11]. Meanwhile, previous studies showed that thermal stratification could influence vertical water quality and cyanobacteria abundance across these thermal layers [8,10]. In our previous studies, a cultural dependent method named BIOLOG was used to explore the effect of thermal stratification on water bacterial community functional diversity, and demonstrated that each layer was known to harbor different bacterial compositions, therefore, thermal stratification could significantly shape bacterial community carbon metabolic characteristics in drinking water reservoirs [4,5]. However, to obtain a more comprehensive understanding of water bacterial communities in deep drinking water reservoirs, investigation on uncultured bacterial phylogenetic community structures across vertical stratified reservoirs is needed [12].

With the development of molecular biological techniques and bioinformatic analysis, Illumina Miseq high-throughput sequencing and multivariate statistical analysis were widely explored to reveal soil [13], sediments [14,15], and wetland [16] microbial community compositions and their relationship with the environmental physicochemical parameters. Recently, Yu *et al.* [17] determined the effects of water stratification and mixing on microbial community structure in a subtropical deep reservoir in southeast China, and suggested that there were significant differences in the water’s physical, chemical and microbiological parameters between epilimnion and hypolimnion layers [17]. Garcia *et al.* [12] also used 454 pyrosequencing to determine the vertical water bacterial composition of a stratified lake, and found that oxygen was an important factor controlling the bacterial community composition. Thus far, very little information regarding the vertical distribution of bacterial community structure in oligotrophic drinking water reservoirs is available.

To this end, the main aim of this work was to determine vertical water quality and bacterial community profiles during the thermal stratification period of micro-polluted reservoirs. The hypothesis was reservoir thermal stratification could significantly influence water quality and shape water bacterial community diversity in the vertical water column. Within this context, in the present study, water quality and bacterial community diversity samples were collected at different depths from JINPEN, a thermal stratified deep drinking water reservoir, and evaluated and mutually compared. The results of this work will give us more information on the relationship between water quality and water microbial diversity during drinking water reservoir thermal stratification conditions.

## 2. Experimental Section

### 2.1. Study Area and Sample Collection

The study was conducted in JINPEN drinking water reservoir, which is located at the foot of Qinling Mountain, Zhouzhi County, Xi’an City, Shaanxi Province, China (34°07′ N, 108°20′ E). JINPEN reservoir is a stratified drinking water reservoir during the summer and autumn and mixing occurred in winter [11]. The maximal depth is 90 m, and average depth is 60 m. The reservoir water volume is 2.0 × 10^8^ m^3^ [2,8,11]. JINPEN reservoir is an important domestic water supply source for Xi’an citizens [8]. Daily water supply capacity for Xi’an is about 8 × 10^5^ m^3^ [8]. As shown in our previous studies, the thermal stratification is formed from May to October each year [8,11]. During the stable stratification period, in June 2013, water samples with three replications at each depth were collected from three typical sampling sites located in the deepest part of the reservoir (GPS positions: 34°02′13.70′′, 108°11′33.92′′; 34°02′39.44′′, 108°11′52.86′′; 34°02′39.81′′, 108°12′20.55′′). In each sampling site, 0.5, 5, 10, 20, 25, 30, 45, 50, 55 and 65 m water depths were collected using a sterilized water sampling device as described previously by our research group [2,8,9,11].

### 2.2. Water Quality Analysis

To better define the water quality properties, water temperature, dissolved oxygen concentration (DO), pH, oxidation reduction potential (ORP), chlorophyll a (*Chl* a), and conductivity were determined using a multi-probe water quality sonde (Hydrolab DS5, Hach, USA) *in situ* [8,11]. Meanwhile, at each depth, a 2 L water sample was collected, loaded in the sterilized bottle, put into the cooler at 8 °C, and transferred immediately to the Environmental Key lab of Shaanxi Province, Xi’an University of Architecture and Technology (XAUAT, Xi’an, China) for further analysis. One liter was used for total nitrogen (TN), nitrate nitrogen (NO_3_-N) and nitrite nitrogen (NO_2_-N) determination. According to the method described by Xie *et al.* [18] and little modification, the concentrations of TN, NO_3_-N and NO_2_-N were examined by a Flow Injection Analyzer (SEAL AA3 Instruments, Norderstedt, Germany) [18].

### 2.3. Water Microbial DNA Extraction

To obtain the total water microbial nucleic acid (DNA), another 1 L sampling of water was filtered with 0.22 μm polycarbonate membranes (Millipore, Billerica, MA, USA). The whole microbial genomic DNA was extracted and purified using Water DNA Kit (OMEGA, Irving, TX, USA) according to the manufacturer’s instructions. The extracted DNA was purified using a DNA purifying kit (DP209 kit, TIANGEN Biotech Co., Ltd, Beijing, P. R. China) and stored at −20 °C for PCR amplification analysis [5].

### 2.4. Illumina Miseq Sequencing and Sequence Analysis

To explore the water bacterial community composition across the vertical thermal stratification layers, an Illumina Miseq Sequencing platform was used in this work. Water bacterial 16S rRNA regions were amplified using PCR Master Mix (KangWei Century Biotech Co., Ltd, Beijing, P. R. China). The primer sets were 515F (5′-GTGCCAGCMGCCGCGG-3′) and 806R (5′-GGACTACHVGGGTWTCTAAT-3′) targeting V4 hypervariable regions of bacterial 16S rRNA genes [16]. 50 μL PCR reactions contained 1 μM of each primer, 0.3 ng of DNA template, 25 μL Master Mix, and pure water (Millipore, Billerica, MA, USA). PCR amplification was at 94 °C for 2 min, 28 cycles of 94 °C for 45 s, 57 °C for 30 s, 72 °C for 30 s, and 72 °C for 5 min [16]. All PCR products were sequenced by an Illumina Miseq Sequencing platform according to standard protocols at Shanghai Majorbio Bio-pharm Technology Co., Ltd, China. After the sequencing process, the raw sequence reads were processed with the MOTHUR software [19]. Sequences with lengths of shorter than 200 bp or low-quality sequences (quality score <25) were removed [20]. After that, the sequencing reads were checked and denoised for chimeras [20]. The taxonomic classification of effective sequences was determined using the Ribosomal Database Project (RDP) database [21].

### 2.5. Nucleotide Sequence Accession Number

Illumina Miseq sequence data were deposited in NCBI sequence read archive (NCBI-SRA) database under the accession number PRJNA23009.

### 2.6. Data Analysis

All statistical analyses were determined on untransformed data using SPSS version 16.0 (SPSS Inc., Chicago, IL, USA). Rarefaction curves (RC), abundance-based coverage estimators (ACE), *Chao*1 richness estimator, the Shannon and Simpson diversity were calculated by MOTHUR (version v.1.21.1) [22]. To examine the relationship between water quality parameters and bacterial community structure diversity, redundancy analysis was employed to reveal water quality properties that had the most significant effects on water bacterial community during thermal stratification period. The RDA figure was constructed by CANOCO software (version 4.5). Heat mapping was performed by *R* package (version 2.1.1) [23].

## 3. Results and Discussion

### 3.1. Vertical Water Quality during the Thermal Stratification

**Figure 1:**
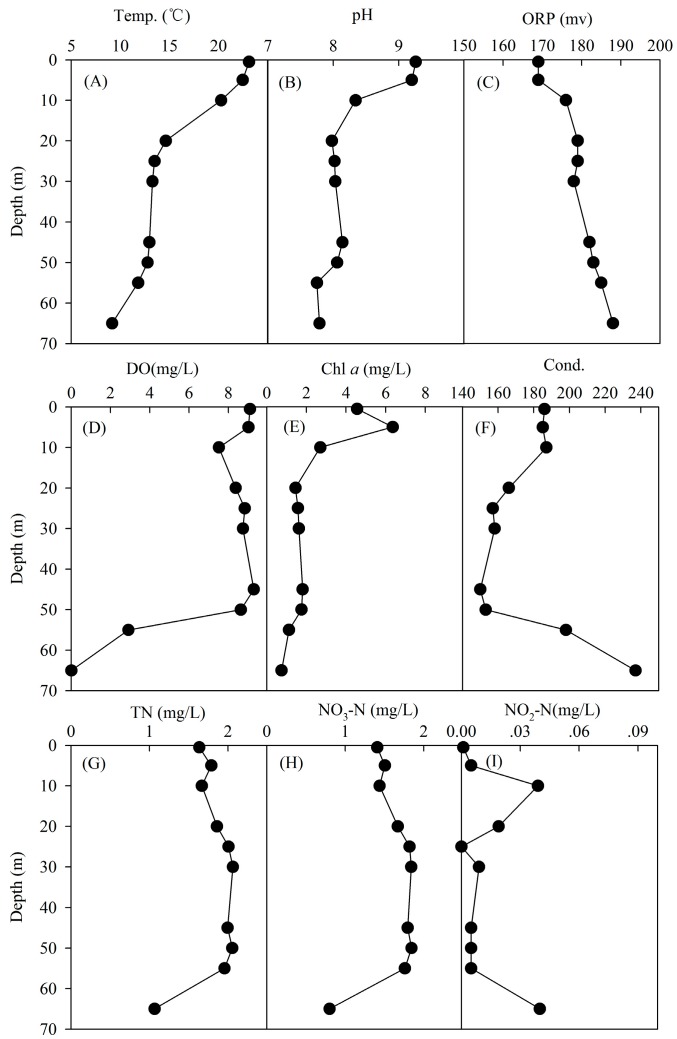
Vertical change of water quality parameters of the JINPEN drinking water reservoir during thermal stratification. Water parameters are including (**A**) temperature (abb. Temp.), (**B**) pH, (**C**) ORP, (**D**) dissolved oxygen, (**E**) chlorophyll a (abb. *Chl* a), (**F**) conductivity (abb. Cond.), (**G**) total nitrogen, (**H**) nitrate nitrogen, and (**I**) nitrite nitrogen.

JINPEN drinking water reservoir is a typical gorge deep reservoir. The maximal depth is 90 m, and average depth is 60 m. As shown in Figure 1, the strongest thermal stratification was formed. Epilimnion, metalimnion and hypolimnion layers were formed steadily in JINPEN reservoir in June 2013. Thermal stratification had significant influence on vertical water quality. There were significant differences in the water’s physical and chemical parameters between epilimnion layer (0.5, 5 and 10 m) and hypolimnion layer (45, 50, 55, 65 m) (*p* < 0.01). The water temperature decreased steadily from the surface to the bottom, ranging from 9.17 to 23.11 °C. Total nitrogen changed from 1.07 to 2.06 mg/L and nitrate nitrogen changed from 0.8 to 1.84 mg/L. The concentration of nitrite nitrogen was very low. The highest conductivity was found in 65 m. The highest chlorophyll a was observed in 5 m, and the lowest in 65 m. The dissolved oxygen concentration decreased sharply below 50 m, and reached zero at 65 m. Our results suggest that physical thermal stratification has a dramatic direct influence on the water quality parameters. This result is consistent with the recent report conducted by Yu *et al.* [17], demonstrating that water temperature, DO, pH and *Chl* a were significantly lower in the hypolimnion than that of epilimnion in Dongzhen reservoir stratification, China [17]. In agreement with the previous report, Ma *et al.* [8] also found the significantly different concentrations of total nitrogen, ammonia nitrogen, and nitrate nitrogen in different layers during the JINPEN drinking water reservoir stratification in 2008. It was revealed that pH decreased from surface to bottom, ranging from 8.7 to 6.2, the highest iron concentration was observed in the bottom hypolimnion layer, ranging from 0.3 to 0.47 mg/L during stratification, and suggested that stratification can induce the endogenous pollution and eutrophication of the JINPEN reservoir [8].

### 3.2. Vertical Water Bacterial Community Diversity during the Thermal Stratification

Using Miseq high-throughput sequencing, after quality trimming, a total of 210,117 sequences with an average length of 435 bp, were obtained for 10 samples (triplicates). As shown in Table 1, in total, the Miseq sequencing revealed a total of 4127 operational taxonomic units (OTUs) with 97% similarity. There are 65,790, 69,075, and 75,252 reads numbers in epilimnion, metalimnion, and hypolimnion layers, and the average reads number in each layer are 21,930, 23,025, and 18,813, respectively. The highest OTUs were found in 5 m, and the lowest was in 0.5 m. The diversity estimator *Chao* 1 is different among depths, ranging from 407 to 506. Shannon diversity is lower in the surface water, and higher in the bottom hypolimnion layer.

**Table 1:** Vertical distribution of water bacterial community diversity and richness estimators of the JINPEN drinking water reservoir during thermal stratification in June 2013.

Thermal Stratification	Water Depths	Reads Number	0.97 Level				
			OTUs	Diversity Estimator		Richness Estimator	
				ACE	*Chao*1	Shannon	Simpson
Epilimnion	0.5 m	15,656	346	402	407	3.76	0.074
	5 m	26,128	476	507	506	4.25	0.038
	10 m	24,006	383	431	430	3.47	0.107
Metalimnion	20 m	17,933	419	475	473	4.10	0.067
	25 m	13,199	401	451	454	4.24	0.046
	30 m	37,943	456	482	482	4.04	0.060
Hypolimnion	45 m	19,711	438	466	462	4.41	0.038
	50 m	35,062	451	474	474	4.16	0.052
	55 m	8282	371	434	443	4.33	0.040
	65 m	12,197	386	445	456	4.03	0.044

Abbreviation: ACE, abundance based-coverage estimator. OTUs, operational taxonomic units. The water bacterial community diversity estimators were calculated on 210,117 reads.

**Figure 2:**
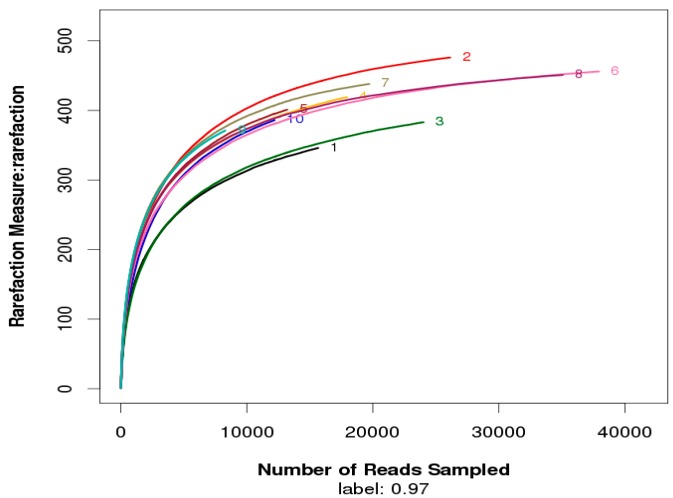
Rarefaction curves of the operational taxonomic units (OTUs) number at 97% similarity boxplot for vertical distribution of bacterial community during JINPEN drinking water reservoir thermal stratification. 1, 2, 3, 4, 5, 6, 7, 8, 9, and 10 represent 0.5, 5, 10, 20, 25, 30, 45, 50, 55, and 65 m depths, respectively.

Rarefaction curves of the OTUs number at 97% similarity boxplot shows that a number of reads were sampled (Figure 2).

**Figure 3:**
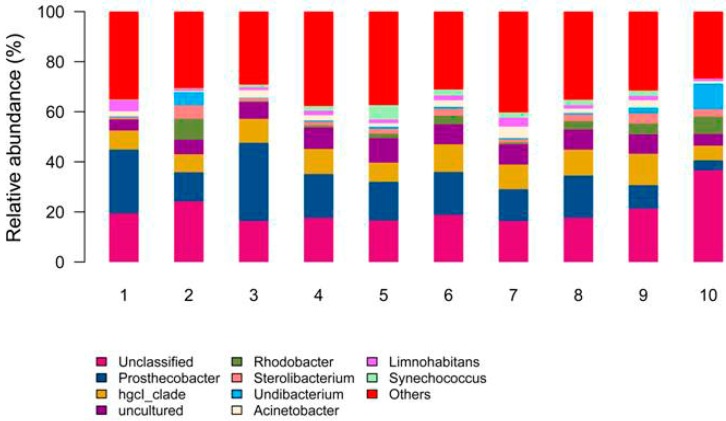
The abundance of dominated bacterial genus in different water layer. 1, 2, 3, 4, 5, 6, 7, 8, 9, and 10 represent 0.5, 5, 10, 20, 25, 30, 45, 50, 55, and 65 m depths, respectively.

The unique and shared operational taxonomic units of bacteria in the vertical depth at 3% distance level showed that in the epilimnion, 251 OTUs were shared at the 0.5, 5, 10 m water layers, meanwhile, in the metalimnion and hypolimnion, 315 and 292 OTUs were also shared at the 20, 25, 30 m, and 45, 50, 55 m water layers, respectively. This suggests that the bacterial community structure in the same stratification layer is stable. The most important explanation for this phenomenon is that water environmental conditions such as DO and pH are not changed significantly. The 4127 operational taxonomic units (OTUs) with 97% similarity were affiliated with 15 phyla including: Acidobacteria, Actinobacteria, Armatimonadetes, Bacteroidetes, Caldiserica, Chlamydiae, Chlorobi, Chloroflexi, Cyanobacteria, Firmicutes, Gemmatimonadetes, Nitrospirae, Planctomycetes, Proteobacteria, and Verrucomicrobia. Meanwhile, as shown in Figure 3, *Prosthecobacter* dominated in the epilimnion layer, *Rhodobacter* and *Undibactrium* dominated in the metalimnion layer, *Synechococcus* dominated in the hypolimnion layer. *Rhodobacter* dominated at 55 m (23.24%) and 65 m (12.58%). *Prosthecobacter* dominated from 0.5–50 m. In agreement with previous literature on stratified reservoirs, bacterial community diversity is significant distinct with depth at the phylum level [17].

**Figure 4:**
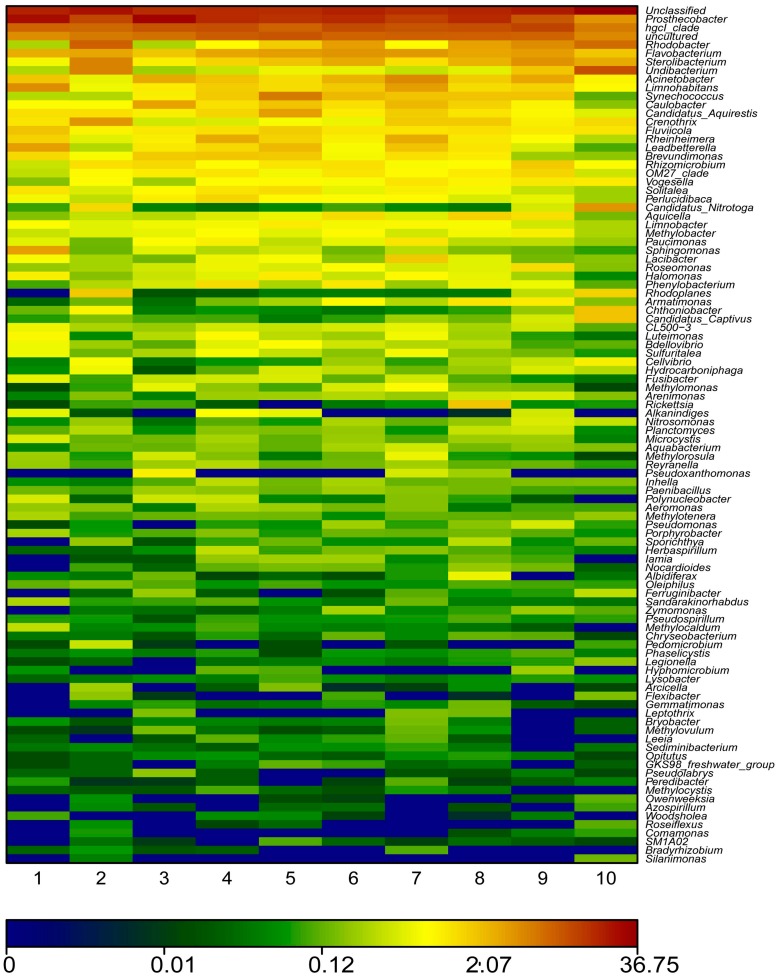
A color-scale heat map showing the top 100 representative predominant 16S rRNA gene-based bacterial sequences classified at the genus level. Red colors indicate higher abundance; blue and green colors indicate lower abundance. 1, 2, 3, 4, 5, 6, 7, 8, 9, and 10 represent 0.5, 5, 10, 20, 25, 30, 45, 50, 55, and 65 m depths, respectively.

**Figure 5:**
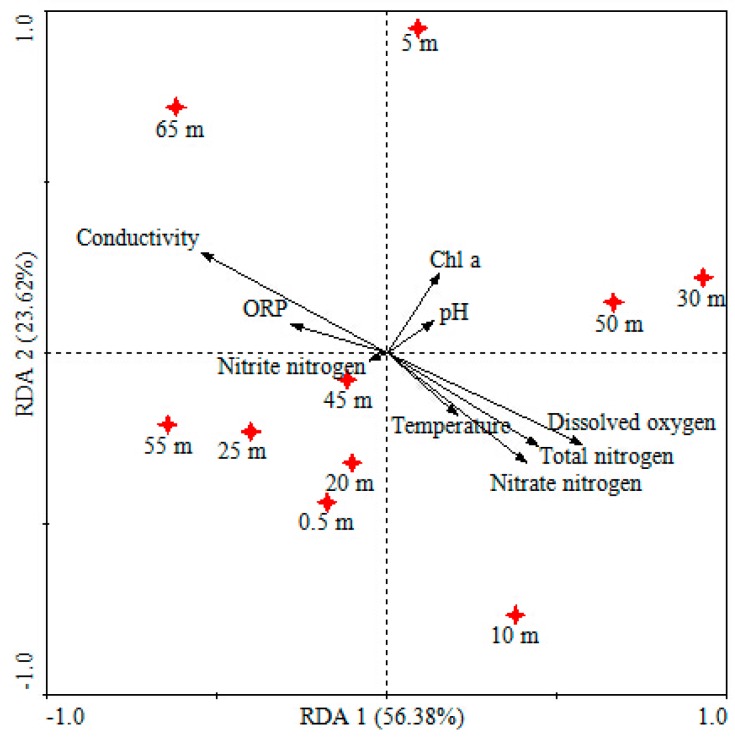
Redundancy analyses of vertical water bacterial communities in the JINPEN drinking water reservoir during thermal stratification. RDA1 explained 56.38%, and RDA2 explained 23.62% of the total variance.

To better understand the effects of water quality on bacterial community diversity, multivariable statistic were employed. As shown in Figure 4 and Figure 5, the heat map fingerprint and redundancy analysis (RDA) indicated significant difference in vertical water bacterial community composition in the reservoir. *Lutermonas* sp., Polynucleobacter, *Methylocaldum* sp., *Woodsholea* sp., and *Sphingonlonas* sp. were observed (Figure 4). The first two RDA dimensions explained 80.0% variation of water bacterial communities. Meanwhile, water quality properties including dissolved oxygen, conductivity, nitrate nitrogen and total nitrogen significantly influenced the water bacterial community compositions (Figure 5).

In the present work, we used Illumina Miseq high-throughput sequencing to explore the vertical characteristics of bacterial communities during JINPEN drinking water reservoir thermal stratification. The results showed that there were significant differences in the water’s physical, chemical and bacterial population parameters between epilimnion and hypolimnion layers. Our previous work also demonstrated that water temperature, dissolved oxygen, and pH may shape water bacterial community metabolic characteristics using BIOLOG technique [2]. Brookes *et al.* [24] reported the effects of diurnal vertical mixing and stratification on phytoplankton productivity in geothermal Lake Rotowhero, New Zealand. In this work, Cyanobacteria were observed in the surface of the JINPEN reservoir. Cyanobacteria have been studied primarily for their morphology as prokaryotic organisms. This result is consistent with our recent microscope observation results [11]. De Hoyos *et al.* [25] determined the distribution and abundance of Cyanobacteria in 47 Spanish water reservoirs during thermal stratification, and 45 taxa of Cyanobacteria were found, suggesting that Cyanobacteria have increased both in biomass and species number in Spanish reservoirs. Messyasz *et al.* [26] also determined *Planktothrix rubescens*, *Synechocystis aquatilis*, and *Desmodesmus grahneisii* diversity in mesotrophic and stratified Lake Holzmaar, Western Germany, revealed that the highest cyanobacterium concentration was detected in the metalimnion, cyanobacterium association was stable in the epilimnion layer, characterized by high temperature, pH values, and oxygen concentration. Similarly, Paganin *et al.* [27] studied the vertical structure of archaeal communities and the distribution of ammonia monooxygenase A gene variants in two Canadian High Arctic lakes, and revealed the dramatically vertical changes of archaeal communities in polar meromictic highly-stratified lakes. Rösel *et al.* [28] studied the free-living water bacterial community in lakes, and suggested that water quality and temperature had important influence on bacterial compositions. A similar study conducted by Comeau *et al.* [29] determined the vertical distribution of bacterial and archaeal communities by high-throughput 16S rRNA gene tag-pyrosequencing in a perennially stratified Arctic lake with saline, anoxic bottom waters, and suggested that bacterial and archaeal communities were stratified by depth from 2 to 60 m. They revealed the relationship between functional species and water parameters gradients. However, our results differ from a study conducted by Shade *et al.* [30], demonstrating that water bacterial communities were resistant to a mixing process. The bacterial communities in the hypolimnion and epilimnion changed due to the mixing, however, water bacterial community can be recovered; hypolimnion and epilimnion layers have the same pattern [30].

During the reservoir thermal stratification period, vertical distribution of water bacterial community is related to water chemistry [30]. In contrast to Yu *et al.* [17], who reported that structural patterns of bacterial communities were related to temperature in the stratified water column in 35 m depth reservoir, we found water dissolved oxygen, conductivity, nitrate nitrogen and total nitrogen significantly influenced bacterial community. This difference could be explained by the fact that the JINPEN reservoir is an oligotrophic drinking water reservoir; the nutrition value is lower than Dongzhen Reservoir (southeast China). Research on carbon and nitrogen cycling in drinking water reservoirs is a hot topic [31,32]. Denitrification bacteria play a vital role in regulating nitrogen cycling in micro-pollution reservoir water systems. In the future, based on high-throughput sequencing combined with stable isotope probing (SIP) techniques [33], further comprehensive studies are needed to accurately examine environmental factors affecting functional genes, such as nitrogen metabolic genes, including *nir*S, *nir*K, *nos*Z, and *amo*A, at different depths in the water column during the summer stratification.

## 4. Conclusions

In conclusion, the present work found that the epilimnion, metalimnion and hypolimnion were formed steadily in the JINPEN drinking water reservoir during thermal stratification periods. The temperature decreased steadily from the surface (23.11 °C) to the bottom (9.17 °C). The dissolved oxygen (DO) concentration decreased sharply below 50 m, and reached zero at 65 m. The Miseq sequencing revealed a total of 4127 operational taxonomic units (OTUs) with 97% similarity, which were affiliated with 15 phyla including Acidobacteria, Actinobacteria, Armatimonadetes, Bacteroidetes, Caldiserica, Chlamydiae, Chlorobi, Chloroflexi, Cyanobacteria, Firmicutes, Gemmatimonadetes, Nitrospirae, Planctomycetes, Proteobacteria, and Verrucomicrobia. The highest Shannon diversity was 4.41 in 45 m, and the highest *Chao* 1 diversity was 506 in 5 m. Reservoir thermal stratification regulated the vertical difference of bacterial community composition. The heat map fingerprint and redundancy analysis (RDA) also indicated significant differences in vertical water bacterial community composition in the reservoir. Meanwhile, water quality properties including dissolved oxygen, conductivity, nitrate nitrogen and total nitrogen dramatically influenced the water bacterial community compositions. The data give a better understanding of oligotrophic drinking water reservoir bacterial community diversity responses to reservoir thermal stratification.

## References

[1] Röske K., Sachse R., Scheerer C. (2012). Microbial diversity and composition of the sediment in the drinking water reservoir Saidenbach (Saxonia, Germany). Systemat. Appl. Microbiol..

[2] Zhang H.H., Huang T.L., Chen S.N., Guo L., Yang X., Liu T.T. (2013). Spatial pattern of bacterial community functional diversity in a drinking water reservoir, Shaanxi Province, Northwest China. J. Pure Appl. Microb..

[3] Röske K., Roske I., Uhlmann D. (2008). Characterization of the bacterial population and chemistry in the bottom sediment of a laterally subdivided drinking water reservoir system. Limnologica.

[4] Zhang H.H., Huang T.L., Chen S.N., Guo L., Yang X. (2014). Microbial community functional diversity and enzymatic activity in the sediments of drinking water reservoirs, Northwest China. Desalin. Water Treat..

[5] Zhang H.H., Huang T.L., Liu T.T. (2013). Sediment enzyme activities and microbial community diversity in an oligotrophic drinking water reservoir, eastern China. PLoS ONE.

[6] Zhao D.Y., Zeng J., Wan W.H., Liang H.D., Huang R. (2013). Vertical distribution of ammonia-oxidizing archaea and bacteria in sediments of a Eutrophic Lake. Curr. Microbiol..

[7] Kagami M., Amano Y., Ishii N. (2012). Community structure of planktonic fungi and the impact of parasitic Chytrids on phytoplankton in Lake Inba, Japan. Microb. Ecol..

[8] Ma Y., Guo Q.L., Huang T.L., Tan P. (2013). Response characteristics of water quality to the seasonal thermal stratification in Jin-pen reservoir along the Heihe river, Xi’an city in China. J. Hydraul. Eng..

[9] Qin C.H., Huang T.L., Li X. (2013). Studies on the seasonal variation and budget of nitrogen, phosphorus of the shibianyu reservoir. J. Xi’an Univ. Architecture Technol..

[10] Elçi Ş. (2008). Effects of thermal stratification and mixing on reservoir water quality. Limnology.

[11] Huang T.L., Li X., Rijnaarts H., Grotenhuis T., Ma W.X., Sun X., Xu J.L. (2014). Effects of storm runoff on the thermal regime and water quality of a deep, stratified reservoir in a temperate monsoon zone, in Northwest China. Sci. Total Environ..

[12] Garcia S.L., Salka I., Grossart H.P., Warnecke F. (2013). Depth-discrete profiles of bacterial communities reveal pronounced spatio-temporal dynamics related to lake stratification. Environ. Microbiol. Rep..

[13] Schmidt P.A., Bálint M., Greshake B., Bandow C., Römbke J., Schmitt I. (2013). Illumina metabarcoding of a soil fungal community. Soil. Biol. Biochem..

[14] Zhang H.H., Huang T.L. (2013). Archaeal community structure and quantity in the oxygen deficient sediments from three water supply reservoirs. J. Pure Appl. Microb..

[15] Wang Y., Sheng H.F., He Y., Wu J.Y., Jiang Y.X., Tam N.F.Y., Zhou H.W. (2012). Comparison of the levels of bacterial diversity in freshwater, intertidal wetland, and marine sediments by using millions of illumina tags. Appl. Environ. Microb..

[16] Jiang X.T., Peng X., Deng G.H., Sheng H.F., Wang Y., Zhou H.W., Tam N.F.Y. (2013). Illumina sequencing of 16S rRNA tag revealed spatial variations of bacterial communities in a mangrove wetland. Microb. Ecol..

[17] Yu Z., Yang J., Amalfitano S., Yu X.Q., Liu L.M. (2014). Effects of water stratification and mixing on microbial community structure in a subtropical deep reservoir. Sci. Rep..

[18] Xie W., Yaoguang Z., Wei Z., Fang X. (2012). The application of flow injection analysis in water quality detection. J. Environ. Hyg..

[19] Schloss P.D., Westcott S.L., Ryabin T., Hall J.R., Hartmann M., Hollister E.B. (2009). Introducing mothur: Open-source, platform-independent, community-supported software for describing and comparing microbial communities. Appl. Environ. Microbiol..

[20] Quince C., Lanzen A., Davenport R., Turnbaugh P. (2011). Removing noise from pyrosequenced amplicons. BMC Bioinform..

[21] Cole J.R., Chai B., Farris R.J., Wang Q., Kulam-Syed-Mohideen A.S. (2007). The ribosomal database project (RDP-II): Introducing myRDP space and quality controlled public data. Nucleic Acids Res..

[22] Loman N.J., Misra R.V., Dallman T.J., Constantinidou C., Gharbia S.E. (2012). Performance comparison of benchtop high-throughput sequencing platforms. Nat. Biotechnol..

[23] Oksanen J., Blanchet F.G., Kindt R., Legendre P., Minchin P.R. (2012). Vegan: Community ecology package. R Package Vers..

[24] Brookes J.D., O’Brien K.R., Burford M.A., Bruesewitz D.A., Hodges B.R., McBride C., Hamilton D.P. (2013). Effects of diurnal vertical mixing and stratification on phytoplankton productivity in geothermal Lake Rotowhero, New Zealand. Inland Waters..

[25] De Hoyos C., Negro A.I., Aldasoro J.J. (2004). Cyanobacteria distribution and abundance in the Spanish water reservoirs during thermal stratification. Limnetica.

[26] Messyasz B., Czerwik-Marcinkowska J., Lücke A., Uher B. (2012). Differences in the ultrastructure of two selected taxa of phytoplankton in a thermally stratified Lake Holzmaar (Germany). Biodiv. Res. Conserv..

[27] Paganin P., Chiarini L., Bevivino A., Dalmastri C., Farcomeni A., Izzo G., Tabacchioni S. (2013). Vertical distribution of bacterioplankton in Lake Averno in relation to water chemistry. FEMS Microb. Ecol..

[28] Rösel S., Allgaier M., Grossart H.P. (2012). Long-term characterization of free-living and particle-associated bacterial communities in Lake Tiefwaren reveals distinct seasonal patterns. Microb. Ecol..

[29] Comeau A.M., Harding T., Galand P.E., Vincen W.F., Lovejoy C. (2012). Vertical distribution of microbial communities in a perennially stratified Arctic lake with saline, anoxic bottom waters. Sci. Rep..

[30] Shade A., Read J.S., Youngblut N.D., Fierer N., Knight R., Kratz T.K., McMahon K.D. (2012). Lake microbial communities are resilient after a whole-ecosystem disturbance. ISME. J..

[31] Comerma M., Garcia J.C., Romero M., Armengol J., Šimek K. (2003). Carbon flow dynamics in the pelagic community of the Sau Reservoir (Catalonia, NE Spain). Hydrobiologia.

[32] Pouliot J., Galand P.E., Lovejoy C., Vincent W.F. (2009). Vertical structure of archaeal communities and the distribution of ammonia monooxygenase A gene variants in two meromictic High Arctic lakes. Environ. Microbiol..

[33] Sun W.M., Cupples A.M. (2012). Diversity of five anaerobic toluene-degrading microbial communities investigated using stable isotope probing. Appl. Environ. Microbiol..

